# Stakeholder perceptions on the deployment of multiple first-line therapies for uncomplicated malaria: a qualitative study in the health district of Kaya, Burkina Faso

**DOI:** 10.1186/s12936-022-04225-3

**Published:** 2022-06-27

**Authors:** Denise Hien, Jean Moise Tanga Kaboré, Mohamadou Siribié, Issiaka Soulama, Nouhoun Barry, Adama Baguiya, Alfred Bewendtaoré Tiono, André-Marie Tchouatieu, Sodiomon Bienvenu Sirima

**Affiliations:** 1 Groupe de Recherche Action en Santé (GRAS), Ouagadougou, Burkina Faso; 2 Swiss Tropical and Public Health Institute, Basel, Switzerland; 3 University of Basel, Basel, Switzerland; 4 Present Address: Internationnal Vaccine Institute (IVI), Seoul, Republic of Korea; 5 Institut de Recherche en Sciences de la Santé (IRSS), Ouagadougou, Burkina Faso; 6 Medicines for Malaria Venture (MMV), Geneva, Switzerland

**Keywords:** Malaria, Perception, Qualitative, MFT, Community, Burkina Faso

## Abstract

**Background:**

In Burkina Faso, malaria remains the first cause of medical consultation and hospitalization in health centres. First-line case management of malaria in the country’s health facilities is based on the use of artemisinin-based combination therapy (ACT). To optimize the use of these anti-malarial drugs in the perspective of mitigating the emergence of artemisinin resistance, which is a serious threat to malaria control and elimination, a pilot programme using multiple first-line therapies (MFTs) [three artemisinin-based combinations—pyronaridine–artesunate, dihydroartemisinin–piperaquine and artemether-lumefantrine] has been designed for implementation. As the success of this MFT pilot programme depends on the perceptions of key stakeholders in the health system and community members, the study aimed to assess their perceptions on the implementation of this strategy.

**Methods:**

Semi-structured interviews, including 27 individual in-depth interviews and 41 focus groups discussions, were conducted with key stakeholders including malaria control policymakers and implementers, health system managers, health workers and community members. Volunteers from targets stakeholder groups were randomly selected. All interviews were recorded, transcribed and translated. Content analysis was performed using the qualitative software programme QDA Miner.

**Results:**

The interviews revealed a positive perception of stakeholders on the implementation of the planned MFT programme. They saw the strategy as an opportunity to strengthen the supply of anti-malarial drugs and improve the management of fever and malaria. However, due to lack of experience with the products, health workers and care givers expressed some reservations about the effectiveness and side-effect profiles of the two anti-malarial drugs included as first-line therapy in the MFT programme (pyronaridine–artesunate, dihydroartemisinin–piperaquine). Questions were raised about the appropriateness of segmenting the population into three groups and assigning a specific drug to each group.

**Conclusion:**

The adherence of both populations and key stakeholders to the MFT implementation strategy will likely depend on the efficacy of the proposed drugs, the absence of, or low frequency of, side-effects, the cost of drugs and availability of the different combinations.

## Background

The World Health Organization (WHO) reported an estimated 229 million cases of malaria in 2019, of which 215 million (94%) were in the African region alone [1]. In Burkina Faso, more than 7 million cases and 14,602 deaths were estimated and the country was ranked among the high burden malaria locations globally; contributing for 4% of all malaria deaths in 2019 [1]. Pregnant women and children under 5 years of age remain the most vulnerable to this disease. Multiple actions are being undertaken to improve malaria prevention through the use of long-lasting insecticidal bed-nets (LLINs), indoor residual spraying (IRS), seasonal malaria chemoprevention (SMC) campaigns, and case management through the adoption of effective ACT recommended by the WHO [2]. In 2005, Burkina Faso introduced artesunate–amodiaquine (ASAQ) and artemether–lumefantrine (AL) as first-and second-line anti-malarial treatments [3]. Several studies have established the efficacy of these therapies [4–8] and the variations between them [9].

Historically, resistance to chloroquine before this millennium emerged and led to the increasing burden of malaria. To respond to this threat, artemisinin-based combinations were developed and recommended by the WHO in countries where malaria parasite is resistant to chloroquine in 2001 [10]. Overuse causes pressure on certain drugs and could shorten their therapeutic life, ultimately leading to resistance [11]. Thus, efforts to preserve the efficacy of the current artemisinin-based combinations are crucial for controlling and eliminating malaria.

To improve the management of malaria, optimize the use of different artemisinin-based combinations, and reduce the risk of emerging resistance, a quasi-experimental pilot research programme using multiple first-line therapies (MFTs), has been planned to make three artemisinin-based combinations (pyronaridine–artesunate, PA, dihydroartemisinin–piperaquine, DHA–PQ, and artemether–lumefantrine, AL) available to public health facilities (PHFs) in the health district (HD) of Kaya for concomitant use in three population groups. Thus, a specific ACT will be allocated to each segment of the population. In support of this pilot research programme, cross-sectional qualitative study was initiated to assess the perceptions of key stakeholders in the health system and community members on the implementation of this MFT strategy. This study was conducted before the roll-out phase of the drugs to the health facilities (HF).

Perception studies are carried out at different stages of the project implementation and aim to produce knowledge, identify stakeholders and their expectations, understand the relationships between groups and take into account all the issues at stake in order to ultimately guide policy creation [12]. These studies articulate several dimensions (social, political and spatial) to understand the phenomenon being analysed [13, 14]. In the field of health research, perception studies make it possible to document the practices of a given population group about a health problem or an innovation in order to take into account the expectations and motivations of direct beneficiaries. Before the adoption of intermittent preventive treatment (IPT) for malaria in children and pregnant women in Burkina Faso and Mali, a perception study helped to understand the enthusiasm of the communities for its adoption [15] and thus reassured the health authorities about its scale-up. In Kenya, before and after the implementation of an intermittent malaria screening and treatment intervention, perception studies identified enabling factors and potential barriers and made recommendations for the likely scaling up of the strategy [16, 17]. Preconceptions about malaria and misperceptions about its management are mostly documented by perception studies of direct users in order to promote appropriate treatment and prevention behaviour [18, 19]. The success of different actions undertaken in favour of populations to reduce malaria-related morbidity and mortality, such as the seasonal malaria chemoprevention (SMC) campaigns, distribution of LLINs, and IRS, depends on the support of the communities [20–24]. Community perceptions of the efficacy of ACT are based on the ability of the drugs to rapidly resolve disease symptoms and provide relief to patients [23–25]. Attitudes and patterns of care-seeking and treatment for malaria provides an understanding of the context in which interventions are delivered [26–29]. Studying and documenting the expectations of different stakeholders with regard to the implementation of the MFT strategy will help to optimize success.

## Methods

### Aim

This study was designed to understand the perceptions and the expectations from different malaria stakeholders, community leaders and caregivers on the implementation of the MFT strategy and to identify potential facilitators and barriers to the district-wide implementation of an MFT pilot programme. It also analysed stakeholders’ perceptions of the causes and prevention of malaria as well as the burden of malaria in the community and health facilities.

### Study setting

This study was conducted ahead of the MFT pilot programme implementation. The MFT programme is a quasi-experimental study aiming at assessing the feasibility, acceptability and costs of a strategy deploying simultaneously multiples first lines therapies for uncomplicated malaria case management. Three artemisinin-based combinations will be deployed at the health facility level. Each of them will be used for the management of uncomplicated malaria in one of the following population groups: PA for children under five, AL for pregnant women and DHA–PQP for individuals aged five years or above. It is being conducted by the Groupe de Recherche Action en Santé (GRAS) in collaboration with the National Malaria Control Programme (NMCP) and the Institut de Recherche en Sciences de la Santé (IRSS).

To generate baseline data on stakeholders’ perceptions and their expectations to inform the MFT programme, this qualitative study was conducted in the HD of Kaya located in the central northern region, 100 km from Ouagadougou in Burkina Faso. It is composed of four communes: one urban (Kaya) and three rural (Pissila, Pibaoré and Mané). It covers an area of 3,617 km^2^ with an estimated population of 365,585 in 2016 [30]. During this study, the district had 40 first-level public health facilities (PHF), including 39 primary care centres and one medical centre with a surgical unit, as well as four private and faith-based health facilities (HF). The referral hospital for the HD is the Kaya Regional Hospital. The HD of Kaya is populated mainly by the Mossi ethnic group and the most widely spoken language in the area is Mooré.

### Study design and populations

This was a qualitative study based on individual in-depth interviews (IDIs) and focus group discussions (FGDs), conducted as a prelude to the implementation of the MFT strategy in the HD of Kaya. It took a holistic approach, taking into account all specific groups within the community, and health systems actors. In Burkina Faso’s health system, each PHF is headed by a nurse and includes a dispensary unit, antenatal and maternal care unit, and generic drugs store. All malaria cases are managed at the dispensary, except for pregnant women. The malaria treatment for pregnant women is done at their specific unit. The generic drug store managers are responsible for the management of supplies, including RDTs and anti-malarial drugs. All of these stakeholder groups were involved in the implementation of the MFT strategy and were considered for inclusion in the sampling for this study. The study population was defined according to the role of each group as malaria control policymakers or as beneficiaries of different malaria control interventions.

#### Individual in-depth interviews—IDIs

The study population for IDIs consisted of respondents taken from stakeholders of the health system in malaria case management who were aged over 18 and had consented to be interviewed. This included key actors at the NMCP, the central purchasing office for essential generic drugs, “Centrale d’Achat des Médicaments Essentiels Génériques” (CAMEG), the regional health directorate and the management team of the HD of Kaya, head of the HFs, and key opinion leaders from the community.

#### Focus group discussions—FGDs

FGDs were conducted with various groups of stakeholders including health care providers (head of HFs, local drug store managers, health workers), vulnerable groups (mothers of children under 5 years of age and pregnant women) and people with decision-making power in the community (community leaders, heads of households, adult men) who could guide and influence their members’ attitudes towards malaria. All participants in the FGDs were over 18 years of age and provided written informed consent to participate in the group discussions.

### Participants selection strategy

Two types of sampling depending on the type of interview and the respondents' profiles were used in this study:

#### IDI

IDIs were conducted at the central and intermediate levels. The respondents were purposively selected according to their role in the national malaria control policy and management of malaria treatment supplies. Health facilities were randomly selected from the list of HFs. The heads of these HFs and the local drug managers were invited to individual interviews. Also, some community leaders were identified within the selected HFs and were invited for individual interviews.

#### FGDs

For the FGDs with different groups of stakeholders: at the HF level, all heads of HFs and all local drug stores managers in PHFs excluding those who participated in the IDIs were included.

The remaining health care providers were randomly selected, allocated by geographical proximity and invited to join in FGDs of 8–12 participants each. At the community level, five HFs were randomly selected and within each HF, participants were randomly selected with the help of community health workers. According to the study protocol [31], the sampling strategies for this qualitative study were described in the Table 1.

**Table 1:** Sampling strategy for qualitative surveys

Target group	Data collection methods and sample size (planned respondents)	Sampling strategy
National malaria control programme	3 IDI	All personnel with key role in malaria case management strategy delivery
Central essential drugs store (CAMEG)	3 IDI	All personnel with key role in ACT ordering and inventory management
North-central health region (Head of the health region, Lead pharmacist, Responsible of the disease control service)	3 IDI	All personnel with key role in malaria case management strategy delivery
Heath district management team of Kaya (Head of the health district, Lead pharmacist Responsible for the district-level healthcare provision)	3 IDI	All personnel with key role in malaria case management strategy delivery
Head of the local health facilities	5 IDI, 4 FGD	5 heads of the local health facilities in the health district of Kaya will be randomly selected and invited for the IDI. The 40 heads of local health facilities will be allocated randomly into for groups for FGD
Essential drugs stores managers at health facilities level	5 IDI, 4 FGD	5 essential drugs store keepers of the local health facilities in the health district of Kaya will be randomly selected and invited for the IDI. The 40 essential drugs store keepers of local health facilities will be allocated randomly into for groups for FGD
Local health workers	10 FGD	10 local health facilities in the health district of Kaya will be randomly selected and the local health workers in charge of malaria case management in pregnant women, children under five and individuals of five years old and above will be grouped for the conduct of the FGD

*ACT* artemisinin-based combination therapy; *FGD* focus group discussion; *IDI* in-depth individual interviews

### Data collection

Semi-structured guides were developed, pre-tested and administered to study participants from 22 January to 29 July 2019. The interviews with health authorities at the central and intermediate levels of the health system were conducted by a senior social scientist.

At the peripheral level, five master’s-level sociology research assistants, fluent in French and the local language (Mooré) carried out interviews and data collection in the field. They were trained on the study protocol, Standard Operating Procedures, conducting IDIs and FGDs, and all data collection tools. This consisted of 2 days of theoretical training and 1 day of practical training which involved testing all materials in the field in French and Mooré. Thus, five pilot testing interviews involving a health worker, a head of association, a traditional chief, a municipal councillor and a mother of a child under five age were conducted to gain information, to identify and address any misunderstanding regarding the interview guides, data collection process, and improve the interview guide’s clarity. All of these interviews were conducted in Polesgo, a peripheral district located in the north of the city of Ouagadougou.

The different people identified to participate in the FGDs were distributed and grouped by municipality (Kaya, Pissila, Mané and Pibaoré) at their nearest HF, to reduce travel distances and increase the participation rate. Each focus group consisted of a minimum of five people and a maximum of 13.

The following key topics were covered in the interview guide according to the profiles of the respondents. With community members, it focused on their knowledge and experiences with malaria, their expectations of the MFT strategy. With health workers, the interviews focused on the burden of malaria and health care management, facilitators and barriers for the implementation of the MFT strategy, and their expectations for the success of this strategy. The interviews guide topics were more focused on the malaria burden and the national policy on malaria management, on the enabling factors and potential obstacles to the success of the MFT strategy with the key health system actors in central and intermediate level. The individual interviews lasted up to 35 min and the FGDs were between 45 and 90 min.

### Data analysis

All interviews were recorded using a digital voice recorder, transcribed in full in French for those conducted in French, or translated from Mooré to French and transcribed in full. Eleven independent social scientist familiar with qualitative data transcribing completed all records transcribing. All transcripts were checked by social scientist (first author), coded and processed using the QDA miner^®^ software. All interviews were coded using a book code by the first author, reviewed by the second author. A thematic analysis of the content of the different interviews helped to design analysis of the perceptions of key actors on malaria and the implementation of the MFT strategy in the Kaya health district. The analysis for this article focuses on the perceptions of key stakeholders on malaria and the implementation of the MFT strategy.

### Ethical considerations and consent to participate

This study received approval from the Ethics Committee for Health Research in Burkina Faso (Clearance no. 2018–8-113/CERS). All interviewed participants were informed about the study and signed an informed consent form before the interviews began. The interview excerpts used in this article do not mention names or anything else that could identify the interview participants. The data output was anonymized to prevent the identification of interview participants.

## Results

A total of 27 IDIs (n = 27 respondents) and 41 FGDs (n = 359 respondents) (Table 2) were conducted with male and female participants aged above 18 years old as shown in the table below. All IDIs were coded 1 to 27 and all FGD 1 to 41.

**Table 2:** IDIs and FGDs conducted with stakeholders

Target groups	IDIs (n = 27 respondents)	FGDs (n = 359 respondents)
NMCP actors	03	00
Central purchasing of essential generic drugs actors	02	00
North-central region health authorities	01	00
The district management team	03	00
Head of HF	05	05
PHF drugs store managers	03	05
Health workers	00	08
Mother of children under 5	00	05
Pregnant women	00	04
Head of household	00	05
Adult men	00	05
Community leaders	10	04
Total	27	41

*FGD* focus group discussion; *IDI* in-depth individual interviews

### Perceptions of malaria and its burden by community members and health workers

#### Community perceptions of malaria

Through the various FGDs and IDIs with community leaders and community members, it was noted their perceptions included that malaria is caused by mosquito bites, the consumption of certain foods, and the practice of domestic animal husbandry, which are a source of insalubrity and wastewater production. While for a long time the causes of malaria were associated with the consumption of certain foods (newly harvested beans, bean leaf sauce, shea butter, very fatty meals, etc.) or contact with rainwater, these concepts are increasingly being relegated in favour of discourse in line with that of modern medicine, according to which malaria is caused by mosquitoes. If the terms ‘*weogo*’ (bush) and ‘*koom*’ (water) are still used by some people to refer to malaria, it is to remember the name of the disease in the local Mooré language, as well as the periods when it is most prevalent. They noted that to protect oneself against malaria, one should: “sleep under insecticide-treated mosquito nets”, “wear long-sleeved clothes”, “keep living areas clean”, avoid “stagnant water”, avoid keeping animals at home, and follow the advice of health workers. The testimonies below illustrate what some of our respondents said about the causes of and prevention tools for malaria.

"If we are in our homes and there is a lot of dirt, we can't stop mosquitoes biting us. For example, we see in our houses where there is sewage, when you go near there, you hear the sound of mosquitoes because that is where they lay and hatch their eggs. We have to make our yards clean. Dirty water, damaged tyres, damaged boxes, every time we have to check and pour the water out. The water in our toilets, we have to clean so that the water doesn't stay there " (IDI24, community leader).

"If the environment is dirty, mosquitoes come from there to bite people. In the rubbish, the dirt, the dirty water causes mosquitoes to bite people.” (FGD17, Mothers of children under 5).

"It is sewage, chicken and animal waste that encourages mosquitoes in the house" (FGD30, pregnant women).

"It is called "weogo" disease. We call it weogo because of the time when it hurts more. Usually in the winter season. During the winter season, when people say they are going to the bush it means they are going into the fields and that’s when the disease is most likely to be triggered. That's what we mean. At the time when you have to invest in fieldwork". (IDI17, community leader).

"We tie up the nets, we advise the women to tie up the nets during the rainy season. When it rains, you have to keep the house clean. The old jars, they should throw them away because they can contain water and stagnant water brings mosquitoes " (IDI15, community leader).

"To protect yourself from malaria, it's like I said, you have to sleep under mosquito nets. Then, in the family, we tell them to avoid dirty water because, it’s there mosquitoes breed [this leader associates mosquito breeding with the presence of sewage in households]. You have to make sure of that [to empty the dirty water]. And you have to keep the houses clean. " (IDI19, Community Leader).

#### Malaria burden in households and health facilities

Through IDIs and FGDs with community members, it was noted that malaria/ “hot body disease” is prevalent in the population, affecting largely children under 5 and pregnant women. Malaria management in HFs is a source of expenditure for households. Community leaders explain it this way:

"The price of drugs [in the public sector] is within the means of the people, it is not so expensive. When we go to buy from the private sector, the price of products there is not the same as in the government generic drug store—it's more expensive [[the private sector]” (IDI11, community leader).

"It [illness] kills. It also reduces people's financial means because when someone is ill you are obliged to stop your activities and look after their health, so it reduces our resources. The fact that he is not in good health there, it is obliged that you leave your work to take care of the health of this person.” (IDI24, community leader).

"The children that are born nowadays are sick, so it's difficult. […] It's a burden. Every time we have to go to the hospital, it is tiring.” (IDI16, community leader).

In the HFs, malaria is still the main reason for medical consultation and hospitalization. Health workers reported they are overworked during the peak malaria periods because of high demand from the population. They also noted that after a consultation for malaria, patients return to the HFs a few days later with the same symptoms, leading health workers to believe there is poor compliance with treatment or that the products prescribed are ineffective. They express it in these terms:

"Malaria is the main reason for consultation, so, normally, it is the one that makes families spend more on care […]; when it comes to complicated malaria, the cost is often high for the target population who are not affected by state subsidies”. (FGD5, head of HF).

"… the majority of patients suffer from malaria. This presents several economic constraints because, as the head of a family, it is your daily work that gives you an income that you use every day to manage your family. And if you have to go 3,4 or 5 days without going to work, it can affect the life of the family. And apart from the economic aspect, on the health itself, it's not easy to live with malaria, it's not easy. It makes you suffer because it's tiring. And people live with it, in fact" (IDI9, key decision-maker, intermediate level).

“We follow their instructions [from healthcare workers]. If you are told to take the medicine in the evening and the morning, we take it according to that. We give the drugs but it [malaria] comes back, it comes back […]” (FG18, mothers of children under 5).

### Stakeholders perceptions about the MFT strategy

The participants’ opinions are based on the explanations and information that the interviewers provided on the strategy, the drugs and the dosage and distribution in the population.

#### The positive perceptions of the MFT strategy by key decision-makers and health workers

For key decision-makers and health workers, the MFT strategy will allow PHFs to have a wide range of anti-malarial drugs that were not previously available to the population. They hope that this strategy will improve the population’s use of PHFs, the quality of care as well as the relationship between health workers (HWs) and patients. Some HWs report misconceptions among some community members about the prescription of AL. Being able to give other drugs will help them to improve the image of the PHF. They explain it as follows:

"There are a lot of advantages because we know that when you use many products at the same time, it allows you to protect all the molecules from emergent resistance.” (IDI3, central level key decision maker).

"The advantage is that we'll have several combinations at least, that's already good, we won't be limited to one combination.” (IDI22, HW).

"We think it's welcome. But only if the programme also manages to do more than we do on the ground. That's it! To manage to raise awareness among the population, that's it, to help the HWs too.” (FGD15, HW).

Speaking about community members' misconceptions about prescribing AL, this health worker explains his experience with a patient as follows:

“We only had Artemether in tablet form. The 6 tablets and the paracetamol tablet. I can assure you that when you prescribe paracetamol and AL tablets, she takes them and comes and throws them in your face like that. […]. When it's a tablet, I know, I can assure you it's not easy, she [the patient] throws it in your face like that, her bag must be full” (FGD12, HW).

The segmentation of the population into three groups will facilitate the use of medicines by community members and prescription by HWs.

“As it is [the drugs are] categorized, when you have a patient in front of you, for example under 5 years old, you know which product to prescribe. So, as long as it is respected, there is no problem.” (IDI10, HW).

"I think it's a good thing because already, here we have three products, if the three products are available, we know that for children it's this, for adults it's this and for pregnant women, it's this. When it's like that, there's no problem, we know how we're going to deal with the population so that we can understand each other.” (FGD5, HW).

### Challenges and fears related to the MFT strategy

Through the IDIs and FGDs, respondents identified some limitations or difficulties. Indeed, some HWs expressed concerns about the relevance of segmenting the population into three groups and assigning a different drug to each segment. They feel that this could make implementation difficult because, in the event of a stock shortage for a given segment, that segment may face difficulties in managing the drug. They will be obliged to resort to other neighbouring health centres or to go to private drugstores. For HWs, the taste and formulation of the study molecules must be in line with the expectations of the population, especially children. The HWs explain their fears related to the MFT strategy. Some quotes are below:

"The problem is that if one type of medicine is not available, you cannot use another type that exists as a substitute. That's a bit of a drawback at this level. There are mechanisms where if artemether–lumefantrine 12 or 6 tablets are not available and if you have the 24 tablets [pack], you can have [dispense] the 6 tablets to treat the patient. But, at this level, there is no full availability. (IDI2, central level key decision-maker).

"There is already this categorization in our daily care. However, as they are the same molecules, this does not pose a problem. For example, when we run out of tablets for adults, we sometimes multiply the number of tablets [meant] for children and give them to adults. So it's easier. But, if you have a shortage in one age group and it's not the same type of drug that you have to use, that can cause a problem. So, it's mainly these aspects. Otherwise, there are no problems” (IDI6, mid-level key informant).

"I take the example of the under 5 s and the over-5 s, […] there are regularly difficulties in sorting things out because some parents will come and insist that their child is over-5 or under-5 just for a particular interest [which may be related to free care]. (IDI6, key decision-maker, intermediate level).

"You have to really emphasize the benefits and also give some side-effects to prepare the population for it. So that they know that even if you take it, a minor effect might be there. But, the beneficial effect is more than the minor effect so that they will not refuse" (IDI5, key decision-maker, middle level).

Generic drugstore managers, on the other hand, believe that the policy of free care has a negative impact on the quality of care and availability of medicines. Because of this policy, some women make several visits to HFs for the same disease, thus accumulating medicines at home and creating stock-outs in drugs store. This could negatively impact the management of ACT during the implementation of the MFT strategy. In their view, the strategy would benefit from selling all drugs at a social price to avoid "waste" and misuse of drugs. They feel that the MFT strategy would also further increase their workload in a context where they feel they are poorly paid. Some drug managers explain their fears in the following:

"Because it's free it's like that. If it was paid for again, they would take care of the medicines. If it's paid for, they'll take care of it, but as they can't afford it, they'll have to be made aware so that they don't waste it because it's free. I have already taken syrups from my home to give to patients at the health centre. When we give them 3 syrups, they use one and put the other two down (FGD25, PHF drugs store managers).

"Several times, we have received patients who have already been in consultations with other health workers before coming here. They come here today, for example on the 19th and you look at their health record and see on the 18th, they were in consultation in another health facility. This means that, with the free service, they do the rounds of the HFs" (IDI8, PHF drugs store managers).

"The addition of products could increase our work, but we will always sacrifice ourselves because it is our population, even if it is not easy. […] We have a lot of notebooks to fill, yet our wages are meagre” (FGD6, PHF drug store managers).

### Perceptions of the MFT strategy by community members

The community leaders hope that the MFT strategy will contribute to better management of malaria and that the different drugs will be used to help make the symptoms of the disease disappear quickly and produce fewer side effects. The single daily dose and the number of tablets per dose would be an asset for the population. Community leaders expressed it in these terms:

"I think it's good. I know that you have gained knowledge [to fight malaria]. The drugs that are there [now], you can take and then see that the disease remains. But, it may be that the medicines that are coming are more effective in relieving [the symptoms and disease]. And because there is a single dose and there are several types, that can be an advantage. Often it's because the drugs are so much to take that people get tired of them. But if it's one pill a day that will cure you, that's good.» (IDI13, community leader).

"In my opinion, the initiative we want to put in place is good. It is good because if we do it like this, the “weogo” disease [malaria] will no longer have the strength to take someone's life in our villages" (IDI16, community leader).

“It can help because the number of medicines to take will decrease. Before, some people could take some of the medicines and leave the rest. But, the fact that the medicines will not be many, they can use them all” (FG32, pregnant women).

This perception of community leaders is largely shared by other community members. Their adherence to the strategy is dependent on the efficacy and tolerability of DHA-PQP and PA. The cost of accessing the drugs is another important factor in determining community adherence to the MFT strategy. For those who benefit from free care, they wanted the strategy to take into account national guidelines for access to health services and care. However, other population groups who do not benefit from free health care hope that the cost of the drugs will be accessible or in line with what was already being done for other anti-malarial.

"Make the medicine free so that people can have the results. Whether it's paid or free, let people have the products [medicines]" (IDI11, community leader).

" We know that this is a good thing. If you had come without going through the health centres, there would be no evidence that it would help us. But the fact that these products are coming in the name of the health centres and not in your name, I think it's a good thing. It is through the health centres that these medicines will reach the people … I don't think it will have many problems. If it has been verified that these are the drugs that will cure our diseases, we will adhere!” (FG27, adult men).

“If it [the medicines] can fight malaria, protect our children, we think it's good. If it can fight disease, it's good” (FG19, Mothers of children under 5).

"I think it will help fight malaria. It will help fight malaria.” (FG20, mothers of children under 5).

The respondents hope for good availability of drugs in drug stores to avoid them moving to other health centres. Dividing the general population into three subgroups and assigning each to one specific malaria drug would be beneficial for people, as it would help them avoid confusion when taking the drugs. They also felt people are different and should not take the same medicines.

“It's good like that, the pregnant women ones apart, the little children apart. It's good because people are not the same. The children don't have enough strength like those who are 5 years old and more.” (IDI13, Community Leader).

Everyone has their own dose of medicine to take. If you are a small child and you take adult medicines, it will make you tired, if an adult also takes children's medicines it is as if he has not taken anything. So we say thank you to God for the medicines that will come there.” (IDI15, community leader).

## Discussion

The purpose of this study was to understand the perceptions of community leaders and key health system stakeholders on the implementation of the MFT strategy for uncomplicated malaria. Most of the people who took part in the IDIs and FGDs identified the mosquito as the main vector of malaria transmission. Although people are aware that the only way to protect themselves from malaria is to sleep under LLINs, many admit that they do not do so. A previous study in Chad had already shown the inconsistency between people's discourse and their practices on the use of LLINs [32]. This attitude of the populations towards the use of mosquitoes and other means of prevention against malaria does not seem to be different within our study population. Indeed, they have a good knowledge of the causes and means of protection against malaria. However, this disease remains the first cause of consultation in health centres.

For community members, mosquitoes thrive in swampy, sewage, stagnant water and in wooded areas. This association between malaria and nature is neither recent nor not specific to this study. The term "malaria" would come from Italian and means "infected area" [33]. This tendency of the population to associate the causes of malaria with the bite of mosquitoes can be associated with the various mass actions undertaken by civil society actors and health workers at community level. This new conception of the disease could explain the choice and therapeutic itineraries of populations seeking malaria treatment.

Concerning the MFT strategy, our study showed that the various key stakeholders have a positive perception, and see it as an opportunity to strengthen the supply of drugs in PHFs for the management of uncomplicated malaria. Indeed, the Ministry of Health of Burkina Faso, through the NMCP, has made available AL and ASAQ for the first-line treatment of malaria to PHFs. However, in the HD of Kaya, patients find the side-effects of ASAQ too hard to bear, NMCP demonstrated the effectiveness of ASAQ in treating uncomplicated malaria [4, 7, 34, 35]. These populations perceived that taking ASAQ causes side effects such as headaches, nausea and dizziness, as described in the package insert. Health workers believe that patients do not follow the instructions for taking ASAQ, which reportedly requires eating a meal before the drug, although this is not mentioned in the package insert. So, to increase compliance with treatment, health workers fall back on AL, for which people have shown a preference.

People's perception of the effectiveness of these drugs is linked to their ability to quickly eliminate the symptoms of the disease and relieve the pain experienced. For community members, the drugs that are deployed as part of the MFT strategy should quickly reduce the symptoms of malaria such as fever and muscle aches and not have too many side effects. The cost of the drugs is also a determining factor for adherence among patients who do not receive free treatment. Although some PHF drug store managers suggest the abolition of free drugs to reduce wastage and stock-outs, several community members and some HWs believe that it is a factor favourable to patient compliance. The cost of medicines has long been an economic burden for poor households, especially those living in rural areas [36].

The interviews also focused on the distribution of ACT by target population group that some community members and health providers found controversial. For some, stock-outs for one target group could affect the continuity of care. Indeed, in the experience of using AL, health facilities had different presentations that they prescribed according to the age and weight of the patients. If one formulation ran out, they could use the other formulations, respecting the dose indicated for the patient according to age and weight. As the MFT programme does not offer such options, this could be problematic for its implementation at the HF level. Other stakeholders appreciated this distribution in the sense that it would avoid the self-medication observed among populations seeking care against fever or malaria. For the latter, the fact that children and adults do not have the same medicines could increase the demand for consultations at the HF level and at the same time reduce the tendency of adults to use the old stock of medicines in case of fever or malaria.

The single daily intake offered by PA and DHA-PQP is appreciated by community members and some health actors. Indeed, AL’s two daily doses have been severely criticized by some community members who found the two doses excessive, and with a taste that they consider unpleasant. Therefore, offering medicines where a single daily dose is recommended will increase compliance. Community HWs are important actors in the management of uncomplicated malaria at the community level. However, the implementation of the MFT strategy will only take place in HFs and might limit the participation of those who seek care to HWs at the community level. As a new strategy, with the introduction of new drugs it was reserved for HFs but this strategy can be extending to the community level depending of the level of understanding of the CHWs and how the health system is organized.

Free health care for children under five and pregnant women increases the access of vulnerable populations to health care [14]. However, that created difficulties in the supply and availability of sanitary inputs. Health providers denounce an “abusive” use of drugs by some members of the community. In addition, refunds are not made within the desired timeframe. This situation creates difficulties for those in charge of health facilities in the procurement and availability of drugs. For some MEG managers, free healthcare is certainly beneficial for the populations, but it impoverishes health centres. The MFT strategy may face the same difficulties.

The limitations of this study lie in its exploratory approach. The participants’ perceptions of ACT are based on their past experiences with other drugs, but also their expectations of what an effective drug should be. Nevertheless, these perceptions are indicative of the expectations of the population and HWs with regard to the management of uncomplicated malaria in the HD of Kaya.

## Conclusion

This qualitative study shows that the key stakeholders of the health system, community leaders and the populations of the HD of Kaya in Burkina Faso have positive perceptions and opinions of MFTs pilot programme. It showed that the population as well as the HWs are in favour of using several combinations as first-line treatments for uncomplicated malaria. As for the MFT strategy, the stakeholders are convinced that it will increase the supply of anti-malarial drugs and improve the management of uncomplicated malaria. The drugs to be deployed in the study are all effective in treating malaria, but HWs and some community members have concerns about potential side-effects. Good communication on possible side-effects while prescribing the drugs could reassure people and increase adherence. Respondents also stressed the preference for once-daily dosing as well as the availability of palatable formulations for paediatric patients. A requirement for administration with food was also listed as undesirable. Free health care has appeared like a potential obstacle to the success of MFTs because of the abusive use of drugs and the long delay in reimbursing the costs associated. Some health care providers perceived filling in the survey documents as extra work that should be paid for.

The attitudes and practices of the population with regard to malaria suggest that many favourable factors exist for the intervention to succeed in the HD of Kaya. Some stakeholders hope that this strategy will reinforce the trust between caregivers and patients. The adherence of the population and health care providers to this strategy is largely based on the effectiveness of DHA-PQP and PA, the control of adverse effects and the continuous supply of generic drugs. This study shows that the MFT strategy will be implemented in a health environment that is favourable to multiple therapeutic combinations with HWs who are aware of and trained in the adequate management of malaria. The population has a good perception of the care provided in HFs and believe that these centres are the appropriate place to treat fever/malaria cases. Despite the favourable environment for MFT implementation, the study drug stock management by HWs and patient education should be considered as a keys component to the success of this new strategy for malaria case management, especially its acceptability.

## Data Availability

The datasets used and analysed during the current study are available from the corresponding author on reasonable request.

## Abbreviations

ACT: Artemisinin-based combination therapy
AL: Artemether–lumefantrine
ASAQ: Artesunate–amodiaquine
CAMEG: Central Purchasing Office for Essential Generic Medicines and Medical Consumables
CHW: Community health worker
DHA-PQP: Dihydroartemisinin–piperaquine
FGD: Focus discussion groups
HD: Health district
HF: Health facility
HW: Health worker
IDI: Individual in-depth interviews
MFT: Multiple first-line therapies
PA: Pyronaridine–artesunate
PHF: Primary health facility
RHD: Regional Health Directorate
SMC: Seasonal malaria chemoprevention
SP: Sulfadoxine–pyrimethamine
WHO: World Health Organization
